# Antimicrobial Resistance in Pathogens Isolated from Blood Cultures: A Two-Year Multicenter Hospital Surveillance Study in Italy

**DOI:** 10.3390/antibiotics10010010

**Published:** 2020-12-24

**Authors:** Francesca Licata, Angela Quirino, Davide Pepe, Giovanni Matera, Aida Bianco, Collaborative Group

**Affiliations:** Department of Health Sciences, School of Medicine, University of Catanzaro “Magna Græcia”, Viale Europa, Germaneto, 88100 Catanzaro, Italy; francesca.licata@studenti.unicz.it (F.L.); quirino@unicz.it (A.Q.); davide.pepe@studenti.unicz.it (D.P.); mmatera@unicz.it (G.M.)

**Keywords:** antimicrobial resistance, hospital, bloodstream infection, surveillance, Multi-Drug Resistance, Italy

## Abstract

Background: Antimicrobial resistance (AMR) is one of the most concerning issues in medicine today. The objectives of this study were to investigate the AMR distribution of the blood-borne pathogens isolated over a two-year period in an Italian region. Methods: A retrospective electronic record review of laboratory-confirmed bloodstream infections (BSIs) was done, and data from three major diagnostic laboratories were used. Twelve invasive clinically important bacteria species were included in the sample. Results: During the study period, 1228 positive BSIs were collected. The most common pathogens were *Coagulase-negative Staphylococcus* (CoNS) (29.7%), *Staphylococcus aureus* (19.1%) and *Escherichia coli* (15.9%). With regard to the AMR pattern, 31.7% of CoNS and 28.1% of *Staphylococcus aureus* were oxacillin-resistant, and almost half of the Enterococci showed resistance to high-level gentamicin. Among Gram-negative species, 11.7% of *Escherichia coli* and 39.5% of *Klebsiella pneumoniae* were carbapenem-resistant. Among the non-fermentative Gram-negative bacteria, the most frequently combined AMR pattern was aminoglycosides and fluoroquinolones (48.4% in *A. baumannii* and 14.6% in *P. aeruginosa*). Conclusion: The results display an alarming prevalence of AMR among hospital isolated pathogens, consistently higher than the European average. Information from surveillance systems to better characterize the trend in the incidence of AMR at local and national levels is needed.

## 1. Introduction

Antimicrobial resistance (AMR) is one of the most concerning issues in medicine today, impacting morbidity, mortality and socio-economic factors [1,2,3], as well as undermining healthcare targets [4]. In hospitals, AMR causes prolongation of stay and increases complication rates and expenditure [5]. In this setting, the emergence and dissemination of multidrug-resistant (MDR) microorganisms, such as methicillin-resistant staphylococci (MRSA) and carbapenem-resistant *Enterobacteriaceae* (CRE), are seriously limiting the options for treatment with antimicrobials, with detrimental effects on survival in patients, especially those with bloodstream infections (BSIs) [6]. The effective management of AMR in hospitals, as well as in the community, is challenging and requires a global strategy. Indeed, no single intervention has proved completely effective in preventing and containing AMR, and the implementation of a package of effective interventions has been advocated [7].

Infections due to MDR microorganisms have triggered the development of coordinated and comprehensive national, European and global actions plans [8,9,10]. As also outlined in the National Action Plan on Antimicrobial Resistance (PNCAR) 2017-2020, monitoring and evaluating interventions requires robust information on the incidence of infections with MDR microorganisms and their impact on the health of populations [8]; however, such information is scarce. Surveillance of the extent and trends of AMR should be established to monitor the process and outcome of containment of AMR [11]. Knowledge of the most likely causative organisms and their expected resistance patterns can increase the probability of selecting an effective drug for empirical treatment [12]. Moreover, appropriate surveillance is essential to monitor resistance trends and help to identify the factors that may be driving them [13]. Such knowledge also provides a baseline for reference as resistance continues to evolve in the future, and represents a prerequisite to implementing rational measures to tackle AMR [14], also at the regional level. Notably, in the hospital setting, constant surveillance of AMR among pathogens is pivotal for advice about variations in local trends and epidemiology [15].

With the foregoing considerations in mind, we decided to investigate the status of AMR by evaluating the distribution and AMR of the blood-borne pathogens isolated over a 2-year period in an Italian region, considering that bacteremia provides a particularly good setting for resistance surveillance [16].

## 2. Results

### 2.1. Sample General Characteristics

Over the entire study period, 10,901 blood samples were cultured and 1228 (11.3%) positive BSIs were reported. The mean age of the patients included in the sample was 60.7 ± 22.8 [standard deviation (SD)] years and the male to female ratio was 1.6:1. The maximum positive BSIs were reported from patients > 65 years of age (54.1%). The majority of isolates were from medical ward patients (41.3%), followed by intensive care unit (ICU) (38.8%), emergency department (ED) (12.9%) and surgical ward (7%) patients. Gram strain classification showed the dominant prevalence of Gram-positive bacteria (57.8%). Among all isolates, bacteria species most frequently found were: Coagulase-negative *Staphylococcus* (CoNS) (29.8%), *Staphylococcus aureus* (*S. aureus*) (19.1%), *Escherichia coli* (*E. coli*) (16%), *Klebsiella pneumoniae* (*K. pneumoniae*) (12%), *Acinetobacter baumannii* (*A. baumannii*) (7.4%), *Enterococcus faecalis* (*E. faecalis*) (5.9%), *Pseudomonas aeruginosa* (*P. aeruginosa*) (3.3%) and *Enterococcus faecium* (*E. faecium*) (2.8%).

### 2.2. Gram-Positive AMR Pattern

The distribution of an antimicrobial-resistant phenotype among Gram-positive organisms by bacterial species and tested antimicrobial agents is reported in Table 1. 

Among CoNS isolates, resistance of >30% was detected towards benzylpenicillin (47.8%), erythromycin (44.8%), gentamicin (39.6%), levofloxacin (38.5%), clindamycin (37.4%) and oxacillin (31.7%). Resistance to vancomycin and teicoplanin was found to be 2.5% and 16.1%, respectively. *S. aureus* strain resistance to benzylpenicillin and oxacillin was 62.1% and 28.1%, respectively. The proportion of *S. aureus* strains resistant to levofloxacin, gentamicin, erythromycin, clindamycin was proximate to 30% (range 29.4–35.3%). Among *Enterococcus species*, high-level gentamicin resistance was shown in 51.4% of *E. faecalis* and 44.1% of *E. faecium*, whereas vancomycin resistance was detected only in one *E. faecium* strain (2.9%). Table 2 reports the distribution of AMR combination among Gram-positive organisms by bacterial species and tested antimicrobial categories. Among CoNS and *S. aureus*, the most common resistance combination was penicillins and aminoglycosides (68.6% and 45.9%, respectively), and penicillins and fluoroquinolones (27% and 23.4%, respectively). An alarming proportion of *Enterococcus* species were resistant both to penicillins and aminoglycosides (38.2% of *E. faecium* and 11.1% of *E. faecalis*) (data not shown in the table).

### 2.3. Gram-Negative AMR Pattern

The distribution of antimicrobial-resistant phenotype among Gram-negative organisms by bacterial species and tested antimicrobial agents is reported in Table 3. 

Antibiograms of *Enterobacteriaceae* displayed an alarming pattern of resistance to ciprofloxacin (46.9% in *E. coli* and 69.4% in *K. pneumoniae*), gentamicin (32.1% in *E. coli* and 52.4% in *K. pneumoniae*) and ceftazidime (30.1% in *E. coli* and 57.1% in *K. pneumoniae*). The proportion of resistance to different carbapenem agents was higher in *K. pneumoniae,* which ranged from 27.9% (ertapenem) to 36.7% (meropenem), than in *E. coli,* which ranged from 6.6% (imipenem) to 8.7% (meropenem). When the category was considered, 39.5% of *K. pneumoniae* and 11.7% of *E. coli* were resistant to at least one agent in the group. A similar pattern was shown for tigecycline (30.6% of *K. pneumoniae* vs 11.2% of *E. coli*) and colistin resistance (18.4% of *K. pneumoniae* vs 8.2% of *E. coli*). Among the non-fermentative Gram-negative bacteria, 58.2% and 56% of the *A. baumannii* isolates were resistant to ciprofloxacin and to gentamicin, respectively; more than a third of the *P. aeruginosa* isolates were resistant to tigecyclin (36.6%), ampicillin (36.6%) and trimethoprim-sulfamethoxazole (36.6%). Cefotaxime resistance was detected in almost half of *A. baumannii* isolates (45.1%) and in one-fifth of *P. aeruginosa* isolates (24.4%). The proportion of resistance to carbapenem agents ranged from 16.5% (ertapenem) to 25.3% (meropenem) for *A. baumannii,* while for *P. aeruginosa*, it ranged from 4.9% (imipenem) to 12.2% (meropenem). The proportion of colistin resistance was proximate to 5% for both *A. baumannii* (5.5%) and *P. aeruginosa* (4.9%). Overall, among the non-fermentative Gram-negative bacteria, the most frequent combined antimicrobial resistance pattern was aminoglycosides and fluoroquinolones (48.4% in *A. baumannii* and 14.6% in *P. aeruginosa*) (Table 4). With regard to *Enterobacteriaceae* resistance combination, 29.6% and 20.9% of *E. coli* and 57.1% and 56.5% of *K. pneumoniae* were resistant to fluoroquinolones and 3rd or 4th generation cephalosporins and to fluoroquinolones and aminoglycosides, respectively. Moreover, resistance to a combination of three categories (3rd or 4th generation cephalosporins, aminoglycoside and fluoroquinolones) was found in 46.9% of the *K. pneumoniae* isolates, and when colistin was also considered, the combined resistance was 15%. 

### 2.4. Binomial Logistic Regression Analysis

The results of the binomial logistic regression models exploring the role of potential predictors of isolated pathogens are presented in Table 5 and show the patient’s age and hospital ward associations. In particular, an increase in age of 10 years results in a 9% reduction in the odds of CoNS isolation (OR = 0.91, 95% CI = 0.86–0.96) and a 10% increase in the odds of positivity to *K. pneumonia* strains (OR = 1.01, 95% CI = 1.01–1.20). With regards to the hospital ward, patients in medical units, compared with those in the ICU, showed higher odds of being affected by *E. coli* (OR = 5.37, 95% CI = 3.34–8.66) and lower probability for CoNS (OR = 0.65, 95% CI = 0.49–0.86), *K. pneumonia* (OR = 0.49, 95% CI = 0.32–0.75) and *A. baumannii* (OR = 0.24, 95% CI = 0.14–0.42). With regards to surgical wards, patients are more likely to be affected by *S. aureus* (OR = 1.79, 95% CI = 1.03–3.12) and *E. coli* (OR = 5.28, 95% CI = 2.72–10.23) compared with those in the ICU. By contrast, the odds are lower for CoNS (OR = 0.21, 95% CI = 0.11–0.43) and *A. baumannii* (OR = 0.15, 95% CI = 0.04–0.64). Finally, the probability to isolate *S. aureus* (OR = 2.00, 95% CI = 1.23–3.27) and *E. coli* (OR = 6.58, 95% CI = 3.63–11.94) was higher among patients in ED compared with those in the ICU; contrarily, it was lower for CoNS (OR = 0.26, 95% CI = 0.15–0.46). The sensitivity analysis conducted as described above did not show any significant alteration of the estimates.

## 3. Discussion

The purposes of this study were to estimate the frequency and distribution of clinically relevant blood–borne pathogens and to estimate their AMR patterns. The data provide valuable information to clinicians and policy makers, and would also be useful to set priorities, within the regions and across the country, and to model future scenarios. 

Similar to previous studies, Gram–positive cocci were the commonly isolated organisms in our study [17,18,19], with CoNS as the most frequent isolated strains [20]. Edmond et al. reported that staphylococci accounted for 47.6% of 10,617 nosocomial episodes of bacteremia [18], and Pittet and colleagues reported that 55% of 1745 nosocomial BSI were caused by Gram–positive cocci [19]. The fact that CoNS are the most common isolates associated with nosocomial BSIs could be related to the wide application of broad–spectrum antibiotics and the increase of invasive procedures that could have changed the etiology of BSI, with the increased detection of CoNS in the recent years. With regard to BSIs caused by Gram–positive cocci, we also found a variation related to the location of patients within the hospital. Indeed, *E. coli* and *S. aureus* species are more likely to be isolated from cultures of blood specimens from non–ICU wards, whereas CoNS are more commonly isolated from the patients in the ICU. A similar location distribution was previously described [21].

Regarding the AMR profile, an important finding was the high percentage of Gram–positive resistance to oxacillin. In the present surveillance, we considered CoNS among the blood–borne pathogens, similar to a previously published study [20]. It should be hypothesized that the oxacillin–resistant CoNS (31.7% in the present study) are mainly restricted to the hospital and differ from more susceptible colonizing strains that commonly contaminate blood cultures. It could be argued that antimicrobial prophylaxis, especially perioperative, could result in the alteration of the cutaneous staphylococcal flora, and documented evidence has suggested that surgical antibiotic prophylaxis has been inappropriately administered and that significant gaps remain with respect to adherence to current evidence–based recommendations in Italian hospitals [22,23]. The proportion of *S. aureus* blood isolates that are resistant to oxacillin (28.1%) was higher than the European Union (EU) Member States’ average (15.5%) [24]. If we take into consideration the Italian situation, the MRSA remains an important pathogen. Although the percentages of resistance have decreased, Italy remains one of the European countries above 30%, with a rate of 35.6% in 2019 [25]. Even higher level of resistance to oxacillin (43.7%) was documented in a previous study conducted in another region of southern Italy [20]. A similar percentage of vancomycin resistance (3.6% vs 3% in the present study) was shown, although the authors documented that *S. aureus* vancomycin resistant strains were absent in the 2015 [20]. It could be argued that the early aggressive administration of broad–spectrum antibiotics has been performed to ensure patient safety. However, maintaining a conservative mindset with respect to antibiotic use is pivotal to reduce hospital stay, cost of therapy and mortality. The authors also underline the fact that the mean age of the patient sample was 60.7 years of age, with 56.5% in the >65 years group. A better analysis of the local and national epidemiological situation investigating the age–group specific incidence is needed, since a previous study found that people aged 55 years or above were mainly affected by invasive MRSA [26]. In Italy, in order to slow down the spread of MRSA, both improved infection prevention and control and prudent antimicrobial use have to be strengthened, as well as monitoring of the zoonotic risk. Another important study finding is the alarming resistance to high–level gentamicin showed by enterococci. It is well–known that these pathogens are naturally resistant or only moderately susceptible to some antibacterials, such as aminoglycosides, and the synergic combination of a cell wall active substance and an aminoglycoside used to treat systemic infections is ineffective in the presence of resistance to one or both components. Moreover, all but one *E. faecium* isolates were not susceptible to vancomycin (2.9%) and all but three to teicoplanin (8.8%). Although the proportion of *E. faecium* that were resistant to vancomycin was lower than that reported in European surveillance report (18.3%) [24], it should be kept in mind that Enterococci are important pathogens, easily disseminated in the healthcare setting, and they represent a high global priority due to the paucity of effective therapeutic options [27].

In our study, the most common Gram–negative organism causing BSI was *E. coli*, which is also implicated as a frequent organism causing BSI in Europe, including Italy [20,24]. With regard to antimicrobial susceptibility pattern among Gram–negative isolates, the highest resistance was observed to fluoroquinolones, aminoglycosides, and 3rd or 4th generation cephalosporins, other than penicillins. Resistance to the last–line group of antibiotics, the carbapenems, was remarkably higher than European resistant strains average for *E. coli* (11.7% vs 0.3%) and *K. pneumoniae* (39.5% vs 7.9%) [24]. A different pattern of antimicrobial susceptibility was reported in a previous study that showed a higher resistance profile to carbapenems of *K. pneumonia*, with an overall resistance level of 65%, but much lower resistance level towards carbapenems of *E. coli* (around 1%) [20]. It seems clear that the levels of CRE have reached hyperendemic levels in Italy. This situation places the national healthcare system at risk by rapidly spreading and exacerbating the already endemic situation in the country. In addition, the loss of effectiveness of this category of antibiotics has led to combinations of antibiotics being used that include molecules which have not been utilized for many years and which are relatively toxic, namely colistin. Unfortunately, *K. pneumoniae* has also exhibited resistance to colistin (18.4%), higher than that reported in a hospital in the same area (4%) [28]. The emergence of colistin resistance has further restricted the number of available drugs, as highlighted by the EuSCAPE project that signaled the detection of pandrug–resistant isolates [29].

In light of the study results, feedback to caregivers about local surveillance data was provided, and specific guidance about the judicious antimicrobial use was supplied to improve antimicrobial choices. In addition, prevention measures for MDR organism infection control were strengthened. Implementation of a “bundle”, i.e., a combination of several proven preventive measures into a single strategy, was launched to prevent central line–associated bloodstream infections, a major focus for the control of MDR organisms. Moreover, the use of active surveillance cultures to detect the “colonization iceberg” of patients with epidemiologically important infectious agents was recommended. The present study has some limitations that deserve comments. First, important information was not captured on the laboratory information system, such as timings of hospital admissions, due to which BSI could not be classified as either hospital or community acquired. Second, due to the retrospective nature of this study, many isolates could not be assessed for molecular studies to evaluate the genetic determinants of AMR. Understanding these factors is surely important, but it goes beyond the aims of this study. Finally, confirmatory AST methods were not performed, as the results presented here were reported as captured on the laboratory information system by diagnostic laboratories.

Even with these limitations, the study findings have the potential to improve patient health and to inform health policies and responses to health threats. Information from active surveillance systems is needed to better characterize the trend in the incidence of AMR at local level, and to examine if consistently decreasing rates of transmission of MDR organisms was achieved after implementation of the recommended control measures. Moreover, the present surveillance data, in some way similar to a comparable regional context [20], despite specific differences, add information about combined antimicrobial resistance pattern of the blood–borne pathogens useful to address specific areas of concern.

## 4. Materials and Methods 

### 4.1. Study Design

A retrospective electronic record review of laboratory–confirmed BSIs was done to describe the AMR among key bacterial microorganisms. The collected data were systematized by their date of isolation, the age and gender of patients and the antibiotic susceptibility profile.

### 4.2. Study Setting

The study was conducted using data from three of the four major diagnostic laboratories in the Calabria region, southern Italy. All these laboratories are accredited to participate in the national passive sentinel surveillance system for AMR (AR–ISS), coordinated by the National Public Health Institute (ISS). The laboratories, one in a teaching hospital and two in large tertiary–care hospitals, collect and send routine AST data to ISS.

### 4.3. Data Source

Records from 1 January 2018 up to 31 December 2019 were analyzed. Twelve invasive clinically important bacteria species were included in the sample as follows: *S. aureus*, CoNS, *E. faecalis*, *E. faecium*, *K. pneumonia*, *E. coli*, *E. cloacae*, *K. aerogenes*, *P. mirabilis*, *S. marcescens*, *P. aeruginosa* and *A. baumannii*. Duplicate isolates, defined as further isolates of the same pathogen with indistinguishable antibiograms obtained from the same patient, were excluded in order to avoid bias due to repetitive sampling of patients undergoing multiple investigations.

### 4.4. Blood Culture and AST

Identification of the bacteria was achieved by culturing aseptically collected paired blood samples in BacT/Alert FAN Plus media bottles and incubated in a BacT/Alert systems (bioMérieux, Florence, Italy). Positive BacT/Alert blood samples were processed for Gram staining and subcultured on solid media (Blood agar and MacConkey agar (bioMèrieux, Florence, Italy)). Colonies were identified using a MALDI–TOF mass–spectrometry technique. The antibiotic susceptibilities of clinical isolates were determined using the automated Vitek^®^2 system (bioMèrieux, Florence, Italy) or BD Phoenix™ Automated Microbiology System (Becton–Dickinson, Heidelberg, Germany) at one laboratory. Final identification and antibiotic susceptibility was performed following the European Committee on Antimicrobial Susceptibility Testing (EUCAST) guidelines and breakpoints (version 7.1–2017) [30].

### 4.5. Statistical Analysis

Data were analyzed using Stata Statistical Software, Version 16. (StataCorp LLC: College Station, TX, USA) [31]. All anagraphic data were anonymized and managed with complete respect of the patients’ privacy, collecting only their gender, age, hospital ward and AST results. Continuous numerical data were expressed as mean ± SD and categorical data were expressed as count and percentages. Binomial logistic regression models have been developed to explore the role of potential predictors of the following isolated species: CoNS (no = 0; yes = 1) (Model 1); *S. aureus* (no = 0; yes = 1) (Model 2); *Enterococcus species* (no = 0; yes = 1) (Model 3); *E. coli* (no = 0; yes = 1) (Model 4); *K. pneumonia* (no = 0; yes = 1) (Model 5); *A. baumannii* (no = 0; yes = 1) (Model 6); *P. aeruginosa* (no = 0; yes = 1) (Model 7). The following variables were included in all models: gender (male = 0; female = 1); age (continuous, years); hospital ward (ICU = 1; Medical wards = 2; Surgical wards = 3; ED = 4). The analysis of standardized residuals was carried out to identify any outliers or any instability of the models. The effect of age was estimated considering the increase of 10 years of age to obtain a more easily interpre-estimate. As a sensitivity analysis, and to avoid unstable estimates, the categories whose cells were represented by fewer than five cases were removed.

### 4.6. Ethics Statement

This is a retrospective study, not directly associated with patients and it was consistent with the principles of the Helsinki Declaration. 

## Figures and Tables

**Table 1:** Distribution of antimicrobial resistant phenotype among Gram-positive organisms by bacterial species and tested antimicrobial agents.

Antimicrobial Category	Antimicrobial Agents	CoNS		*Staphylococcus aureus*		*Enterococcus faecalis*		*Enterococcus faecium*		Total	
		N = 366	(%)	N = 235	(%)	N = 72	(%)	N = 34	(%)	N = 707	(%)
Penicillins	PEN	175	(47.8)	146	(62.1)	14	(19.4)	19	(55.9)	354	(50.4)
	OXA	116	(31.7)	66	(28.1)	NT		NT		182	(30.3) ^1^
Fluoroquinolones	CIP	52	(14.2)	39	(16.6)	12	(16.7)	8	(23.5)	111	(15.7)
	LVX	141	(38.5)	77	(32.8)	28	(38.9)	12	(35.3)	258	(36.5)
Aminoglycosides	GEN	145	(39.6)	78	(33.2)	- ^2^		- ^2^		223	(37.1) ^1^
	HLG	NT		NT		37	(51.4)	15	(44.1)	52	(49.1) ^1^
Macrolides	ERY	164	(44.8)	83	(35.3)	23	(31.9)	10	(29.4)	280	(39.6)
Lincosamides	CLI	137	(37.4)	69	(29.4)	- ^2^		- ^2^		206	(34.3)^1^
Tetracyclines	TET	88	(24)	43	(18.3)	12	(16.7)	7	(20.6)	150	(21.2)
Folate pathway inhibitors	SXT	96	(26.2)	53	(22.6)	- ^2^		- ^2^		149	(24.8) ^1^
Glycopeptides	VAN	9	(2.5)	7	(3)	0		1	(2.9)	17	(2.4)
	TEC	59	(16.1)	33	(14)	6	(8.3)	3	(8.8)	101	(14.3)
Oxazolidinones	LZD	18	(4.9)	7	(3)	0		0		25	(3.5)
Lipopeptides	DAP	23	(6.3)	20	(8.5)	7	(9.7)	0		50	(7.1)

Abbreviations: CoNS, Coagulase-negative *Staphylococcus;* CIP, ciprofloxacin; CLI, clindamycin; DAP, daptomycin; ERY, erythromycin; GEN, gentamicin; HLG, high-level gentamicin; LVX, levofloxacin; LZD, linezolid; OXA, oxacillin; PEN, benzylpenicillin; SXT, trimethoprim-sulfamethoxazole; TEC, teicoplanin; TET, tetracycline; VAN, vancomycin; NT: not tested. ^1^ Percentages are calculated among the total tested organisms. ^2^ According to the Clinical & Laboratory Standards Institute CLSI, *Enterococcus species* susceptibility to low-level aminoglycoside, clindamycin and trimethoprim-sulfamethoxazole should not be reported, as they may appear active in vitro but are ineffective clinically.

**Table 2:** Distribution of the antimicrobial resistance (AMR) combination among Gram-positive organisms by bacterial species and tested antimicrobial categories.

AMR Pattern ^1^	CoNS		*S. aureus*		Total	
	N = 366	(%)	N = 235	(%)	N = 601	(%)
**Resistance to two antimicrobial categories**						
Penicillins + Aminoglycosides	120	(68.6)	67	(45.9)	187	(31.1)
Penicillins + Fluoroquinolones	99	(27)	55	(23.4)	154	(25.6)
Aminoglycosides + Fluoroquinolones	80	(21.9)	32	(13.6)	112	(18.6)
**Resistance to three antimicrobial categories**						
Penicillins + Aminoglycosides + Fluoroquinolones	71	(19.4)	29	(12.3)	100	(16.6)
Penicillins + Aminoglycosides + Glycopeptides	30	(8.2)	17	(7.2)	47	(7.8)
Penicillins + Fluoroquinolones + Glycopeptides	27	(7.4)	19	(8.1)	46	(7.6)
**Resistance to four antimicrobial categories**						
Penicillins + Aminoglycosides + Fluoroquinolones + Glycopeptides	24	(6.6)	15	(6.4)	39	(6.5)

^1^ Resistance was defined as non-susceptibility to ≥ 1 agent in the considered antimicrobial categories.

**Table 3:** Distribution of the antimicrobial resistant phenotype among Gram-negative organisms by bacterial species and tested antimicrobial agents.

Antimicrobial Category	Antimicrobial Agent	*E. coli*		*K. pneumoniae*		*A. baumannii*		*P. aeruginosa*		Others Gram-Negative Organisms ^1^		Total	
		N = 196	(%)	N = 147	(%)	N = 91	(%)	N = 41	(%)	N = 46	(%)	N = 521	(%)
Penicillins	AMP	79	(40.3)	91	(61.9)	44	(48.4)	15	(36.6)	11	(23.9)	240	(46.1)
Penicillins + β-lactamase inhibitors	AMX	66	(33.7)	91	(61.9)	9	(9.9)	5	(12.2)	20	(43.5)	191	(36.7)
	TZP	33	(16.8)	61	(41.5)	17	(18.7)	8	(19.5)	11	(23.9)	130	(25)
2nd generation cephalosporins	CXM	21	(10.7)	12	(8.2)	3	(3.3)	-		-		36	(8.3) ^2^
	FOX	16	(8.2)	35	(23.8)	20	(22)	2	(4.9)	3	(6.5)	76	(14.6)
3rd or 4th generation cephalosporins	CTX	53	(27)	83	(56.5)	41	(45.1)	10	(24.4)	20	(43.5)	207	(39.7)
	CAZ	59	(30.1)	84	(57.1)	8	(8.8)	7	(17.1)	19	(41.3)	177	(34)
	CPM	31	(15.8)	46	(31.3)	15	(16.5)	5	(12.2)	13	(28.3)	110	(21.1)
Aminoglycosides	AMK	17	(8.7)	62	(42.2)	27	(29.7)	4	(9.8)	12	(26.1)	122	(23.4) ^2^
	GEN	63	(32.1)	77	(52.4)	51	(56)	9	(22)	14	(30.4)	214	(41.1)
Fluoroquinolones	CIP	92	(46.9)	102	(69.4)	53	(58.2)	9	(22)	24	(52.2)	280	(53.7)
Carbapenems	IMP	13	(6.6)	45	(30.6)	16	(17.6)	2	(4.9)	5	(10.9)	81	(15.5)
	MEM	17	(8.7)	54	(36.7)	23	(25.3)	5	(12.2)	10	(21.7)	109	(20.9)
	ETP	14	(7.1)	41	(27.9)	15	(16.5)	4	(9.8)	7	(15.2)	81	(15.6)
Glycylcyclines	TGC	22	(11.2)	45	(30.6)	14	(15.4)	15	(36.6)	8	(17.4)	104	(20)
Folate pathway inhibitors	SXT	50	(25.5)	59	(40.1)	39	(42.9)	15	(36.6)	16	(34.8)	179	(34.4)
Polymyxins	CST	16	(8.2)	27	(18.4)	5	(5.5)	2	(4.9)	8	(17.4)	58	(11.1)

Abbreviations: AMX, amoxicillin-clavulanic acid; AMK, amikacin; AMP, ampicillin, CTX, cefotaxime; CAZ, ceftazidime; CIP, ciprofloxacin; CST, colistin; SXT, trimethoprim-sulfamethoxazole; CPM, cefepime; CXM, cefuroxime and cefuroxime axetil; ETP, ertapenem; FOX, cefoxitina; GEN, gentamicin; IMP, imipenem; MEM, meropenem; TZP, piperacillin/tazobactam; TGC, tigecycline; NT: not tested. ^1^ *Proteus mirabilis*, *Klebsiella aerogenes*, *Enterobacter cloacae*, *Serratia marcescens*. ^2^ Percentages are calculated among the total tested organisms.

**Table 4:** Distribution of the AMR combination among Gram-negative organisms by bacterial species and tested antimicrobial categories.

AMR Pattern ^1^	*E. coli*		*K. pneumoniae*		*A. baumannii*		*P. aeruginosa*		Others Gram-Negative Organisms ^2^		Total	
	N = 196	(%)	N = 147	(%)	N = 91	(%)	N = 41	(%)	N = 46	(%)	N = 521	(%)
**Resistance to two antimicrobial categories**												
Carbapenems + Aminoglycosides	20	(10.2)	53	(36.1)	22	(24.2)	4	(9.8)	9	(19.6)	108	(20.7)
Carbapenems + Fluoroquinolones	19	(9.7)	56	(38.1)	21	(23.1)	3	(7.3)	10	(21.7)	109	(20.9)
3rd or 4th generation cephalosporins + Aminoglycosides	31	(15.8)	72	(49)	13	(14.3)	3	(7.3)	11	(23.9)	130	(25)
3rd or 4th generation cephalosporins + Fluoroquinolones	58	(29.6)	84	(57.1)	12	(13.2)	5	(12.2)	17	(37)	176	(33.8)
Aminoglycosides + Fluoroquinolones	41	(20.9)	83	(56.5)	44	(48.4)	6	(14.6)	16	(34.8)	190	(36.5)
**Resistance to three antimicrobial categories**												
Carbapenems + Aminoglycosides + Fluoroquinolones	18	(9.2)	51	(34.7)	20	(22)	3	(7.3)	9	(19.6)	101	(19.4)
3rd or 4th generation cephalosporins + Aminoglycosides + Fluoroquinolones	27	(13.8)	69	(46.9)	11	(12.1)	3	(7.3)	11	(23.9)	121	(23.2)
**Resistance to four antimicrobial categories**												
Carbapenems + Aminoglycosides + Fluoroquinolones + Polymyxins	3	(1.5)	17	(11.6)	1	(1.1)	0	(-)	0	(-)	21	(4)
3rd or 4th generation cephalosporins + Aminoglycosides + Fluoroquinolones + Polymyxins	2	(1)	22	(15)	1	(1.1)	0	(-)	2	(4.3)	28	(5.4)

^1^ Resistance was defined as non-susceptibility to ≥ 1 agent in the considered antimicrobial categories. ^2^ *Proteus mirabilis*, *Klebsiella aerogenes*, *Enterobacter cloacae*, *Serratia marcescens*.

**Table 5:** Results of binomial logistic regression analysis.

Blood Culture Isolates	Gender ^1^		Age (10 Years Increase)		Wards ^2^					
					Medical Wards		Surgical Wards		Emergency Rooms	
Outcome	OR	95% CI	OR	95% CI	OR	95% CI	OR	95% CI	OR	95% CI
CoNS	1.12	0.85–1.46	0.91	0.86–0.96	0.65	0.49–0.86	0.21	0.11–0.43	0.26	0.15–0.46
*S. aureus*	1.14	0.84–1.54	1.05	0.98–1.13	1.42	1.01–2.00	1.79	1.03–3.12	2.00	1.23–3.27
*Enterococcus* species	1.01	0.66–1.55	1.02	0.92–1.12	1.37	0.84–2.24	1.40	0.62–3.16	1.82	0.92–3.59
*E. coli*	1.08	0.77–1.50	1.08	0.98–1.18	5.37	3.34–8.66	5.28	2.72–10.23	6.58	3.63–11.94
*K. pneumoniae*	0.85	0.58–1.24	1.01	1.01–1.20	0.49	0.32–0.75	1.65	0.94–2.90	0.57	0.29–1.09
*A. baumannii*	0.70	0.42–1.16	0.97	0.89–1.06	0.24	0.14–0.42	0.15	0.04–0.64	NE	NE
*P. aeruginosa*	0.85	0.41–1.77	1.00	0.84–1.18	2.03	0.88–4.70	NE	NE	2.79	0.93–8.32

NE = Not estimable due to empty regression cells. ^1^ Reference category = male gender. ^2^ Reference category = Intensive Care Unit.

## Data Availability

The data presented in this study are openly available in MendeleyData repository (doi:10.17632/jd8p77cb9n.1).
